# Lupus tumidus: a report of two cases*

**DOI:** 10.1590/abd1806-4841.20164940

**Published:** 2016

**Authors:** Bianca Pinheiro Bousquet Muylaert, Bruna Backsmann Braga, Eduarda Braga Esteves, Luciana Elisa Barandas Garbelini, Alexandre Ozores Michalany, Jayme de Oliveira Filho

**Affiliations:** 1 Universidade de Santo Amaro (UNISA) – São Paulo (SP), Brazil

**Keywords:** Antimalarials, Autoimmune diseases, Lupus erythematosus, cutaneous

## Abstract

Lupus tumidus is considered a rare subtype of chronic cutaneous lupus erythematosus, characterized by erythema and bright urticarial erythematous-violaceous lesions that leave no scars after regression. Histopathology reveals perivascular and periannexal lymphohistiocytic infiltrates in the papillary and reticular dermis and interstitial mucin deposition. Treatment is based on photoprotection, topical corticosteroids and antimalarials. We report two cases of lupus tumidus, which deserve attention for their low frequency in the literature, in addition to their relevance as a differential diagnosis among dermatologic disorders.

## INTRODUCTION

Lupus erythematosus is a multisystem, heterogeneous, autoimmune disease characterized by the production of autoantibodies against cellular constituents. The most affected organ is the skin, which may be involved in isolation or accompanied by systemic manifestations.^1,2^ It primarily affects young women between 18 and 30 years regardless of racial group. Genetic, environmental, socio-cultural and demographic differences may contribute to differing incidences as well as the clinical expression of the disease.^1,2^

Cutaneous manifestations of lupus can be classified into specific subtypes – which include chronic cutaneous lupus erythematosus (CCLE), subacute cutaneous lupus erythematosus (SCLE) and acute cutaneous lupus erythematosus (ACLE) – and nonspecific skin lesions – such as panniculitis, vasculitis and tumid lesions.^1^ However, some authors disagree with this classification claiming that tumid lupus should be considered a separate entity from other forms of lupus, as it shows important responses to treatment with antimalarial drugs, extreme photosensitivity and characteristic histopathologic findings.^3^

Lupus tumidus was first described by Gougerot and Bournier in 1930.^4^ Clinically, it is characterized by shiny erythematous-violaceous urticarial lesions in sun-exposed areas that leave no scars.^2^

Histopathologically, the epidermis is generally spared or presents discrete focal vacuolar degeneration of the basal membrane, associated with a perivascular lymphohistiocytic lesion infiltrated in the papillary and reticular dermis and interstitial mucin deposition.^1,2^ Direct immunofluorescence is usually negative in the dermoepidermal junction.^5^

## CASE REPORT

**Figure 1:**
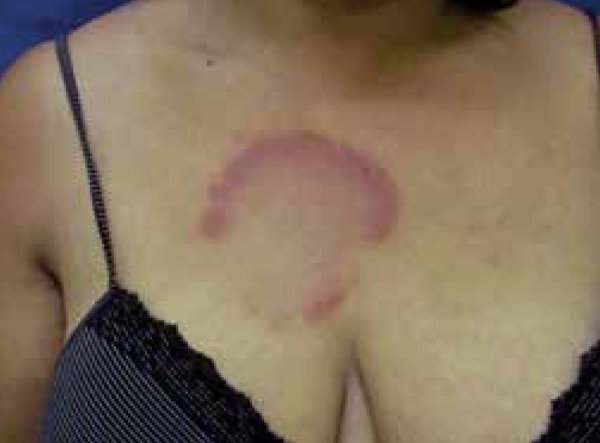
Erythematous infiltrated lesion in the thorax

**Figure 2:**
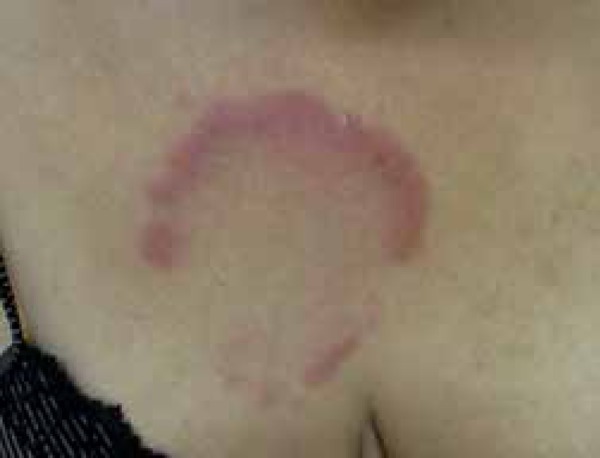
Erythematous infiltrated lesion in the thorax with central clearing

A 50-year-old female patient presented with a complaint of lesion on the thorax for 4 months. She reported an erythematous macule at the anterior region of the trunk, which evolved into an erythematous infiltrated lesion with central clearing (Figures 1 and 2).

**Figure 3:**
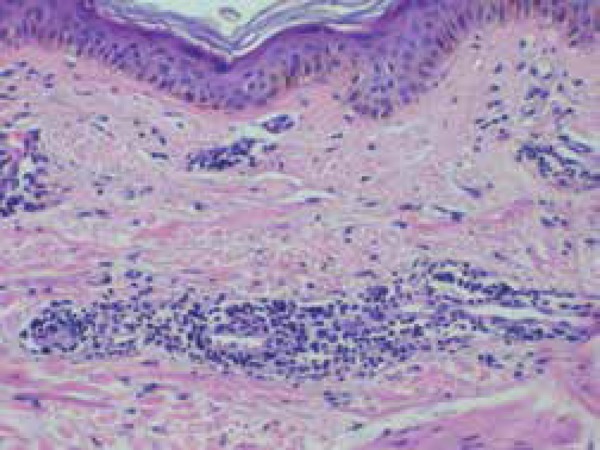
Hematoxylin-eosin: atrophy of the spinous layer, numeric and volumetric reduction of interpapillary ridges in the epidermis. Moderate perivascular infiltrate in the dermis

**Figure 4:**
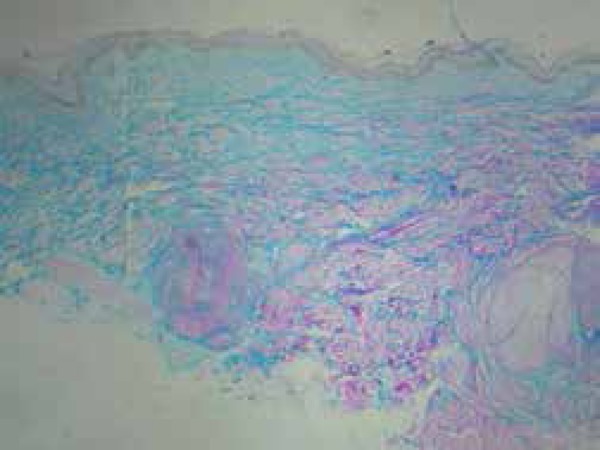
Colloidal iron stain: collagen fibers are dissociated by mucin accumulation

We performed an incisional biopsy and stained the sample with hematoxylin-eosin, colloidal iron and Ziehl-Neelsen. Pathologic study revealed a moderate predominantly perivascular lymphocytic infiltrate not affecting the blood vessels in the papillary dermis and superficial and deep reticular dermis (Figure 3). Collagen fibers were separated by mucin accumulation, which was confirmed by colloidal iron staining (Figure 4). Epidermis showed atrophy of the spinous layer and numerical and volumetric reduction of interpapillary ridges.

Clinical and histopathological findings confirmed our hypothesis of lupus tumidus. We excluded the possibility of associated systemic lupus after laboratory tests (only anti-DNA antibody was moderately reagent) and introduced treatment with antimalarial drug – chloroquine 250mg/day – and guidance about photoprotection. The patient responded well to treatment and we observed no new lesions.

**Figure 5:**
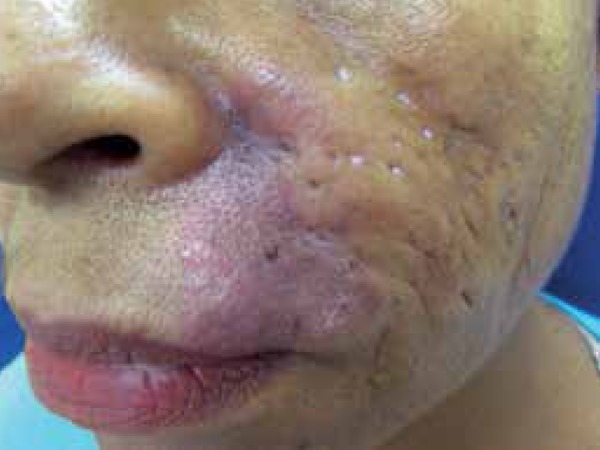
Erythematous-infiltrated plaque on the nasolabial sulcus

**Figure 6:**
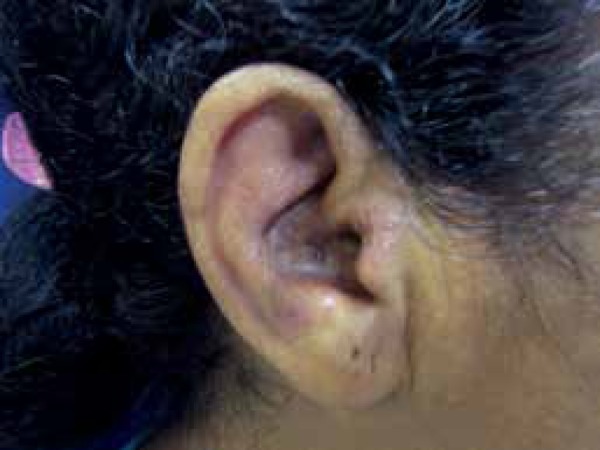
Erythematous-infiltrated plaque on the right ear

Another 50-year old female patient reported a one-year history of erythematous-infiltrated plaques – 10cm in size – on the nasolabial sulcus and on the right ear (Figures 5 and 6). She also mentioned the appearance of a small erythematous-infiltrated plaque (about two centimeters in diameter) at the left frontoparietal region fifteen days before the medical appointment. Biopsies of the skin on the left frontal region and of the left nasolabial sulcus suggested the diagnosis of lupus tumidus. Anti-nuclear antibody, anti-Ro antibody, complete blood count and complement tests were all within the normal range. Treatment consisted of oral prednisone, chloroquine, and topic hydrocortisone lotion. The patient remains under prednisone 5mg/day and chloroquine 250mg/day for the past 5 months without any new skin lesions.

## DISCUSSION

Chronic cutaneous lupus erythematosus (CCLE) has polymorphous presentations that may occasionally mimic other clinical conditions, causing diagnostic difficulties. Lupus tumidus is a rare subtype of CCLE, and its diagnosis can be confirmed by the correlation between clinical and histopathological manifestations.^6^

The diagnosis of lupus tumidus is usually delayed, as it can be confused with other dermatoses due to the absence of systemic manifestations.^3^ Kuhn et al. (2000) proposed the following criteria for lupus tumidus diagnosis: clinical and histological results, reproduction of lesions after exposure to UVA and/or UVB and quick and effective response to treatment with antimalarial drugs. The clinical criteria is met with the presence of erythematosus, thick plates with a smooth and edematous surface on sun-exposed areas that leave no scar after regression. Histological signs are perivascular and periadnexal lymphocytic infiltrate, interstitial mucin deposition and, in some cases, diffuse lymphocytes, or absence of epidermal involvement or any change in the dermoepidermal junction.^5^

Lupus tumidus differs in several aspects from the other variants of CCLE, such as the absence of scars when involuted, epidermal atrophy and follicular plugging and adherent hyperkeratotic scaling, all of which are present with discoid lupus. Unlike subacute cutaneous lupus erythematosus (SCLE), lupus tumidus presents no residual hypopigmentation. Histopathology reveals no follicular hyperkeratosis, epidermal atrophy, vacuolar degeneration or basal membrane thickening, usually identified at SCLE and discoid lupus.^5^

The main differential diagnosis is Jessner’s lymphocytic infiltration, which is clinically expressed as asymptomatic papulonodular lesions affecting sun-exposed areas that last for several months and leave no scars when regressing. However, unlike lupus tumidus, histopathology examination reveals no interstitial mucin deposition in Jessner’s lymphocytic infiltration. The disease fills the diagnostic criteria for lupus tumidus proposed by Kuhn, which justifies the controversy in the medical literature that question whether Jessner is a lupus tumidus variant or if it is an autonomous entity. Reticular erythematous mucinosis (REM) – a primitive form of mucinosis that worsens with sun exposure and presents satisfactory response to treatment with antimalarial drugs - should also be listed in the differential diagnosis. Despite the histopathologic similarities to lupus tumidus, the lymphocytic infiltration is usually less dense and mucin accumulates mainly at the papillary dermis in REM. The main difference between the diseases is the clinical manifestation.^3^

We suggest systemic antimalarial drugs as the treatment of choice for lupus tumidus. Kind et al. first described effective results with that treatment in 1992. The treatment can also include topical corticosteroids, systemic corticosteroids and high sun protection factor levels (30or higher).^5^ Recently, Kuhn et al. described a treatment with photodynamic therapy as an alternative, but it is unable to prevent lesion recurrence.^7^

Due to the rarity of cases reported, we emphasize the importance of detailed clinical examination supplemented by histopathological study since isolated examination may lead to underdiagnosing the disease.
